# Supplementary material for: Type II Heterojunction Formed between {010} or {012} Facets Dominated Bismuth Vanadium Oxide and Carbon Nitride to Enhance the Photocatalytic Degradation of Tetracycline
Source: Int J Environ Res Public Health. 2022 Nov 10;19(22):14770. doi: 10.3390/ijerph192214770 (PMC9690978; doi:10.3390/ijerph192214770)
Supplement: Supplementary file 1 [file ijerph-19-14770-s001.zip › ijerph-1981706-Supplementary.pdf]

Supporting Information

Type II heterojunction formed between {010} or {012} facets dominated bismuth vanadium oxide and carbon nitride to enhance the photocatalytic degradation of tetracycline

Xiaojing Zhang [a,+], Xianglun Xie [a,+], Jianan Li [a], Dongfang Han[a], Yingming Ma [a*], Yingying Fan [a*], Dongxue Han [a,b], and Li Niu [a,b]

[a] *Center for Advanced Analytical Science, Guangzhou Key Laboratory of Sensing Materials & Devices, School of Chemistry and Chemical Engineering, Analytical and Testing Center, Guangzhou University, Guangzhou 510006, PR China;*

[b] *Guangdong Provincial Key Laboratory of Psychoactive Substances Monitoring and Safety, Anti-Drug Technology Center of Guangdong Province, Guangzhou 510230, PR China;*

[*] *Correspondence: ccymma@gzhu.edu.cn; ccyyfan@gzhu.edu.cn*

[+] *These authors contributed equally to this work.*

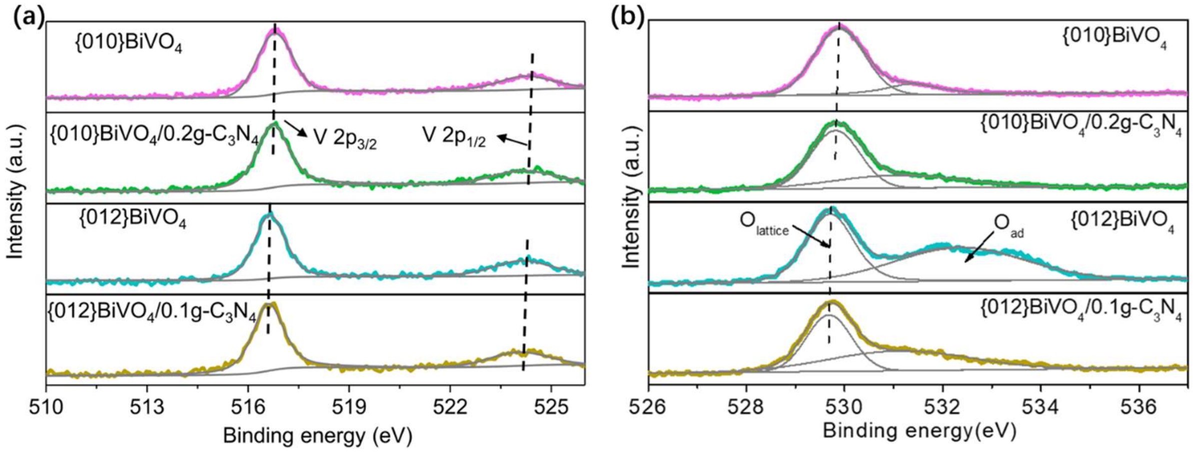

Fig. S1 High resolution XPS spectra of (a)V2p and (b) O1s on {010}$BiVO_4$, {010}$BiVO_4$/0.2g-C_3N_4, {012}$BiVO_4$ and {012}$BiVO_4$/0.1g-C_3N_4.

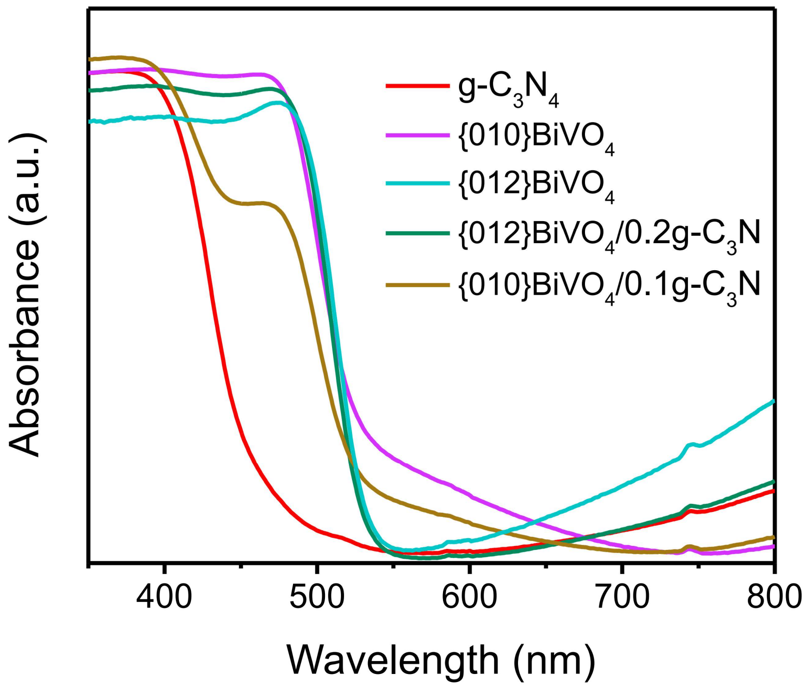

Fig. S2 UV-Vis diffuse reflectance spectra of g-C_3N_4, {010}$BiVO_4$, {012}$BiVO_4$, {010}$BiVO_4$/0.2g-C_3N_4 and {012}$BiVO_4$/0.1g-C_3N_4.

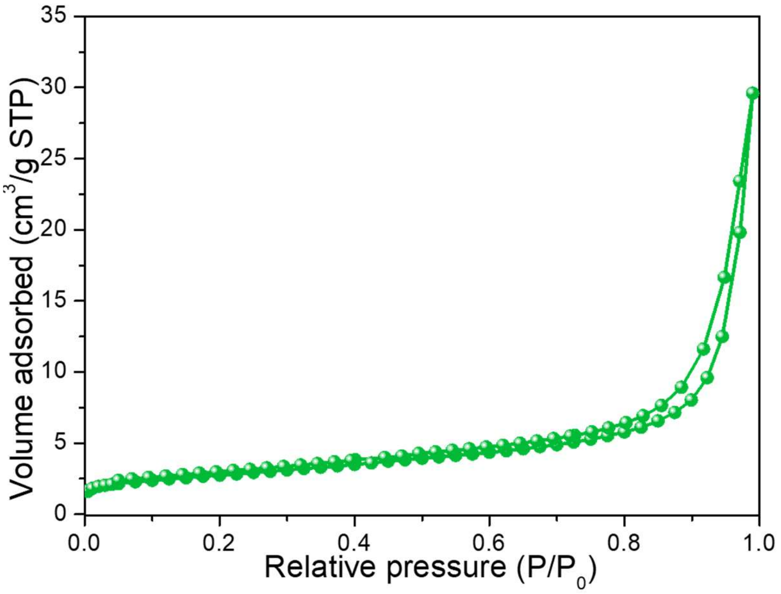

Fig. S3 N_2 adsorption-desorption isotherms over {010}$BiVO_4$/g-C_3N_4.

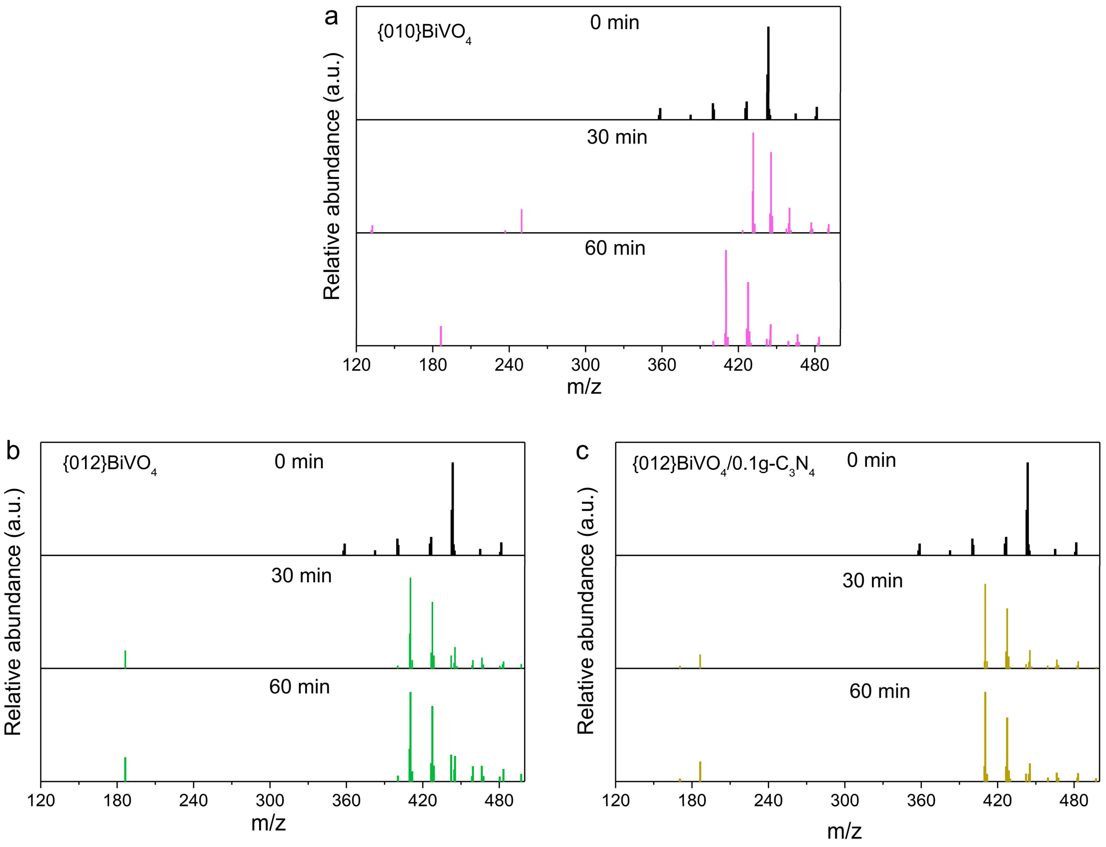

Fig. S4 LC-MS spectra of intermediates over (a) {010}$BiVO_4$, (b) {012}$BiVO_4$ and (d) {012}$BiVO_4$/0.1g-C_3N_4 during TC degradation.
